# Factors Promoting Mental Health of Adolescents Who Have a Parent with Mental Illness: A Longitudinal Study

**DOI:** 10.1007/s10566-015-9304-3

**Published:** 2015-02-07

**Authors:** L. M. A. Van Loon, M. O. M. Van De Ven, K. T. M. Van Doesum, C. M. H. Hosman, C. L. M. Witteman

**Affiliations:** 1 Behavioural Science Institute, Radboud University Nijmegen, P.O. Box 9104, 6500 HE Nijmegen, The Netherlands; 2 Community Mental Health Centre Dimence, Deventer, The Netherlands

**Keywords:** Protective factors, Problem behavior, Parental mental illness, Adolescence

## Abstract

**Background:**

Children of parents with mental illness have an elevated risk of developing a range of mental health and psychosocial problems. Yet many of these children remain mentally healthy.

**Objective:**

The present study aimed to get insight into factors that protect these children from developing internalizing and externalizing problems.

**Methods:**

Several possible individual, parent–child, and family protective factors were examined cross-sectionally and longitudinally in a sample of 112 adolescents. A control group of 122 adolescents whose parents have no mental illness was included to explore whether the protective factors were different between adolescents with and without a parent with mental illness.

**Results:**

Cross-sectional analyses revealed that high self-esteem and low use of passive coping strategies were related to fewer internalizing and externalizing problems. Greater self-disclosure was related to fewer internalizing problems and more parental monitoring was related to fewer externalizing problems. Active coping strategies, parental support, and family factors such as cohesion were unrelated to adolescent problem behavior. Longitudinal analyses showed that active coping, parental monitoring, and self-disclosure were protective against developing internalizing problems 2 years later. We found no protective factors for externalizing problems. Moderation analyses showed that the relationships between possible protective factors and adolescent problem behavior were not different for adolescents with and without a parent with mental illness.

**Conclusions:**

The findings suggest that adolescents’ active coping strategies and parent–child communication may be promising factors to focus on in interventions aimed at preventing the development of internalizing problems by adolescents who have a parent with mental illness.

## Background

Survey studies in the Netherlands, Australia, and Norway have reported that between 17.0 and 37.3 % of the children in the general population have a parent with mental health issues (Goossens and Van der Zanden 2012; Maybery et al. 2009; Torvik and Rognmo 2011). Children of parents with mental illness, hereafter referred to as COPMI, have an elevated risk of developing internalizing and/or externalizing problems themselves, for example anxiety or depression, or aggressive behavior. Several empirical studies have reported that the risk of these children to develop problems is two to even thirteen times higher than is the risk of children of parents without psychological problems (Beardslee et al. 1993; Dean et al. 2010; Weissman et al. 2006). In spite of the increased risk, not all COPMI develop psychological difficulties. In fact, many of these children remain mentally healthy (Gladstone et al. 2006). It is important to understand which factors *protect* COPMI from developing psychosocial problems. Understanding the protective factors is important when designing preventive interventions that could help these children at risk.

The interest in a strengths-based empowerment approach in mental health in both research and practice is growing (Simon et al. 2005). Such research has focused on protective factors to promote resilience especially among children living in high-risk conditions. The present study aimed to examine factors that protect children against developing problem behavior, as these factors can be easily included in interventions for mental health promotion and prevention purposes.

In the present study, we focused on adolescents, where most previous studies on COPMI focused on young children (Beardslee et al. 2011; Goodman et al. 2011). Adolescence is an important developmental period in which rates of psychological problems, such as depression, increase significantly (Graber and Sontag 2009). Most adolescents go through this phase without major problems (Steinberg 2011), but adolescent COPMI may encounter difficulties due to, for instance, insufficient emotional support from their parents (Roustit et al. 2010). We want to examine several possible protective factors in this risk group to identify those that could strengthen and protect them. In addition, we want to examine whether these protective factors are specific for adolescent COPMI by comparing the relationships between possible protective factors and problem behavior of adolescents with and without a parent with mental illness, in order to contribute to the provision of tailored preventive interventions for the at-risk youth.

Protective factors can be examined on an individual, a dyadic (parent–child), and a broader family level. An important individual factor is the adolescent’s *coping strategy.* Coping can be defined as ‘conscious volitional efforts to regulate emotion, cognition, behavior, physiology, and the environment in response to stressful events or circumstances’ (Compas et al. 2001, p. 89). Active coping strategies that deal directly with the stressor (e.g., confronting the problem) are usually related to positive outcomes, whereas passive coping strategies, such as avoiding the problem, are mostly related to negative outcomes (Meijer et al. 2002). For example, in a general population of adolescents, it was found that higher avoidance was related strongly to more depressive symptoms (Dumont and Provost 1999). Studies examining coping strategies of adolescents with a depressed parent (e.g., Jaser et al. 2008; Langrock et al. 2002) have shown that secondary control coping (i.e., cognitive restructuring, positive thinking, acceptance, distraction) was related to fewer symptoms of anxiety/depression and aggressive behavior problems. However, these studies of adolescents with a depressed parent did not use the active versus passive coping approach; thus, it makes it difficult to compare them with the studies in the general adolescent population. While these studies examined coping responses specifically related to parental depression, the present study aimed to examine coping strategies of adolescents whose parent has one or more of a broader range of mental illnesses, including depression. Some cross-sectional studies have been done with adolescents (albeit sometimes using different coping concepts), but no previous studies have longitudinally examined the effect of coping strategy on problem behavior in a sample of adolescent COPMI.

Adolescent *self*-*esteem* is another possible protective individual factor we focused on in this study. In general adolescent populations, high self-esteem has been found to be related to fewer internalizing problems, such as depressive symptoms (Dumont and Provost 1999), and fewer externalizing problems, such as aggression, antisocial behavior, and delinquency (Donnellan et al. 2005). A decade ago, Baumeister et al. (2003) pointed out the lack of longitudinal studies on the effect of self-esteem on (mental) health outcomes. Since then, several prospective studies have been conducted with a general population of adolescents. For example, low self-esteem during adolescence was found to predict poor mental health outcomes and a higher risk of being convicted of a crime during adulthood (Trzesniewski et al. 2006). Enhancing self-esteem is also an important aim of several prevention programs targeting COPMI, who often feel isolated from peers and whose self-esteem might consequently suffer (Orel et al. 2003). However, to our knowledge, no previous studies have examined the (prospective) relationship between self-esteem and internalizing and externalizing problems of adolescent COPMI.

Of the possible protective factors on a dyadic level, parent–child interaction has been shown to be an important predictor of positive outcomes for vulnerable children (Rutter 1990). Parent–child interaction can have many dimensions. Parental monitoring and parental support are two factors that have consistently been identified as predictors of positive outcomes in adolescence (e.g., Barnes and Farrell 1992; Kerr and Stattin 2000). *Parental monitoring* has been described as “parents’ knowledge of the child’s whereabouts, activities, and associations” (Stattin and Kerr 2000, p. 1074), and more parental monitoring has been associated with fewer internalizing (Jacobson and Crockett 2000) and externalizing (Patterson 1993) problems in adolescence. The negative relationship between parental monitoring and externalizing problems has also been shown among adolescent COPMI (Van Loon et al. 2014). Because the latter study was cross-sectional, it does not make clear whether parental monitoring protects these at-risk youth against developing negative outcomes later.

*Parental support* has been defined as ‘emotional availability’ (Duncan and Reder 2000) and ‘parental nurturance’ (Elgar et al. 2007). Parental support is related to fewer internalizing (Roustit et al. 2010) and externalizing problems (Stice et al. 1993; Wills and Cleary 1996) in adolescence. Brennan et al. (2003) showed that perceived maternal warmth and acceptance were associated with resilient outcomes (such as no current internalizing problems) in a sample of adolescents with a depressed mother. However, their study was cross-sectional, and the effect of parental support on the development of internalizing and externalizing problems over time was not assessed.

Another parent–child interaction factor that has repeatedly been related to adolescent well-being is adolescents’ *self*-*disclosure towards parents*. There is an inverse relationship between how much adolescents tell their parents and their norm-breaking behavior (Stattin and Kerr 2000) and delinquency (Soenens et al. 2006). Self-disclosure has mostly been examined in relation to externalizing problems in adolescence, but Finkenauer et al. (2002) have examined it in relation to internalizing problems. They found greater self-disclosure towards parents to be associated with fewer physical complaints and less loneliness, but no significant relationship was found with depressive mood. No previous studies have examined the protective effect of self-disclosure on the development of both internalizing and externalizing problems longitudinally, and neither has the concept of self-disclosure, to our knowledge, been examined in a sample of adolescent COPMI.

Apart from dyadic (parent–child) family factors, it is also important to consider the broader family environment (i.e., parents and siblings). *Family cohesion* (i.e., commitment between family members) was found to be associated with fewer internalizing and externalizing problems in adolescence (Barber and Buehler 1996). A previous study has examined the protective value of family cohesion in a sample of adolescents who have a parent with substance abuse problems (Farrell et al. 1995). This study found that low family cohesion was related to more deviance, distress, and heavy drinking in adolescents. However, there are no previous studies that have examined the protective value of family cohesion in families with a broader range of mental illnesses. The opportunity to openly and directly express emotions and opinions within the family (“*family expressiveness*”) might be an important protective factor as well, although previous research is inconclusive about the relationship with different adolescent outcomes. Some studies found that less family expressiveness was related to adolescent delinquency (Bischof et al. 1995) while others found no relationship with a different adolescent outcome, namely depression (Cole and McPherson, 1993). *Family conflict* also plays an important role in adolescence, as conflict between parents and children usually increases during this period (Steinberg 2011). High family conflict has been associated with negative mental health outcomes in adolescence, such as depressive feelings (Fendrich et al. 1990).

The last family factor we focused on in the present study is *perceived family support*, defined as a subjective feeling of support from the adolescents’ next of kin (e.g., parents, siblings, grandparents, uncles, aunts, cousins). In a sample of high-stress adolescents, it was found that those who felt high family support reported fewer internalizing problems (i.e., depression) and externalizing problems (i.e., delinquency) (Licitra-Kleckler and Waas 1993). To our knowledge, no study to date has examined whether perceived family support is related to and predictive of fewer internalizing and externalizing problems in adolescent COPMI.

## Current Study and Hypotheses

Although there is an extensive body of literature about factors that protect adolescents in the general population against problem behavior, much remains to be known about the prospective influences of specific individual, dyadic (parent–child) and broader family factors on internalizing and externalizing problems of adolescents who have a parent with mental illness (COPMI). The current study extended previous research in several ways. First, it focused on adolescent COPMI, a group that has so far received little attention. Second, it not only used cross-sectional but also longitudinal data of two waves (i.e., a period of 2 years). Finally, a control group of adolescents who have no parents with mental illness (non-COPMI) was included, allowing us to evaluate which factors are specifically important for families with a parent with a mental illness.

This study investigated the following questions: (1) Which individual, dyadic (parent–child), and broader family factors are related to internalizing and externalizing problems of adolescent COPMI at baseline? (2) Which individual, dyadic (parent–child), and broader family factors at baseline protect adolescent COPMI against the development of internalizing and externalizing 2 years later?, and (3) Are these abovementioned relationships different for adolescent COPMI and a control group of adolescent non-COPMI? We hypothesized that high self-esteem, high use of active coping strategies, and low use of passive coping strategies would be related to and protective of developing internalizing and externalizing problem behavior by adolescent COPMI. Furthermore, we expected that better parent–child interaction (i.e., more parental support, more parental monitoring, and greater self-disclosure) and a positive family environment (i.e., more cohesion, more expressiveness, less conflict, and more perceived family support) would be related to and predictive of a decrease in internalizing and externalizing problems of these vulnerable adolescents. Given the exploratory nature of the third research question, no specific hypotheses about differences or similarities between adolescent COPMI and non-COPMI in these (prospective) relationships were tested.

## Methods

### Sampling and Procedure

The ethics committee of the Faculty of Social Sciences of the Radboud University Nijmegen approved the protocol of the study. The study included adolescent children who have a parent with mental illness (COPMI) and a comparison group of adolescents who have no parent with mental illness (non-COPMI). Non-probability sampling was utilized for this study. Families with an adolescent child and a parent who has mental illness were recruited through different channels. First, general practitioners’ access to parental diagnoses was gained through the data from the ‘Registration Network General Practitioners (RNGP) Limburg’ (Metsemakers et al. 1992). Patients with (1) currently active codes of depression, anxiety disorder, and/or alcoholism based on the ‘International Classification of Primary Care’ (ICPC) and (2) children aged 11–16 were selected and mailed a letter informing them about the goals and procedures of the study and asking for their participation. At time 1, 32 families recruited via GPs met the inclusion criteria and returned the questionnaires. The second way to contact eligible families was through mental health institutions. Therapists were asked to give an invitation letter to clients who (1) were diagnosed with depression, anxiety disorder, and/or alcohol related problems and (2) had children aged 11–16. At time 1, nine families recruited via mental health institutions met the inclusion criteria and returned the questionnaires. The third recruitment strategy involved approaching potential participants via advertisements in local newspapers and over the Internet by asking parents with (1) a mental illness and (2) a child between 11 and 16 years to participate in our study. Five families recruited via advertisements met the inclusion criteria and completed the questionnaires at time 1. Fourth, schools were approached and asked to distribute letters about the study to their 11–16 years old pupils. In this letter, parents suffering from a mental illness were asked to participate. If both parent and child were willing to participate, they could contact us via the website or by e-mail or telephone. At time 1, 98 families recruited via schools met the inclusion criteria and returned the questionnaires. The final recruitment strategy was to contact families who had participated in a previous study (Van Santvoort et al. 2014) where (1) at least one parent suffered from a mental illness and (2) the children were in the appropriate age range (11–16 years). Twenty-nine of these families met the inclusion criteria and participated in the study. The families with parents without a mental illness (i.e., the control group) were recruited through GPs and schools (58 and 132 families, respectively, met inclusion criteria and completed the questionnaires). Families completed the written registration form enclosed with the recruitment letter or an online registration form to register for the study. Inclusion and exclusion criteria were checked in a telephone call. Children with significant developmental delay and/or a severe chronic illness were excluded, as were families who were not able to complete the questionnaires due to language problems. Consent forms were sent to the families by mail along with the parent and child questionnaires. Two years later, parents and adolescents completed the paper-based questionnaires again. The families received a monetary reward (€10 for T1, €30 for T2) for their participation after they had returned the questionnaires by mail.

Per family, one parent and one child participated in the study. When there were more children in the 11–16 years age range, we selected the oldest child by default, unless there were other reasons, such as significant developmental delay, to select a younger brother or sister. In the COPMI families, at least one parent had a mental illness. Parental mental illness status was validated through self-report of the parent (i.e., parent answered ‘yes’ on the single item ‘Do you have mental health complaints?’), and confirmed by either a mental health professional or by a score above the cut-off level on one of several well-validated questionnaires (HADS: Hospital Anxiety and Depression Scale, Zigmond and Snaith 1983; GHQ: General Health Questionnaire, Goldberg 1972; CAGE: problem drinking questionnaire, Ewing 1984) (see Table 1). The HADS assesses feelings of depression and anxiety, with a score of 8 or higher on (one of) the subscale(s) indicating at least mild mood and/or anxiety disturbance (Snaith and Zigmond 2000). For the CAGE, a cut-off score of two was used, with two or more positive answers suggesting the likelihood of having alcohol related problems (Ewing 1984). For the GHQ, a cut-off score of three or higher was used to identify people likely to have mental health problems (Goldberg and Williams 1988). Families with no parents with a mental illness (1) did not self-report mental illness and (2) had scores below the cut-off levels on the mental health questionnaires (see Table 1).

**Table 1:** Criteria for being assigned to the group of families with a parent with mental illness or of families with parents without mental illness, by recruitment strategy

	PMI	No PMI
Recruitment strategy		
General practitioners	1. Active ICPC code for depression/anxiety/alcohol related problems	1. No active ICPC code for any psychological problem
	2. Self-report of mental health problems/HADS ≥ 8/CAGE ≥ 2	2. No self-report of mental health problems
		3. HADS ≤ 7, CAGE ≤ 1, GHQ ≤ 1
Mental health institutions	1. Mental health problems confirmed by professional	–
	2. Self-report of mental health problems	
Advertisements	1. Self-report of mental health problems	–
	2. HADS ≥ 8/CAGE ≥ 2/GHQ ≥3 /confirmation professional	
Schools	1. Self-report of mental health problems	1. No self-report of mental health problems
	2. HADS ≥ 8/CAGE ≥ 2/GHQ ≥ 3/confirmation professional	2. HADS ≤ 7, CAGE ≤ 1, GHQ ≤ 1
Previous studyª	1. Mental health problems confirmed by professional	–
	2. Self-report of mental health problems	

PMI, parent with mental illness; no PMI, no parents with mental illness; ICPC, international classification of primary care; HADS, Hospital Anxiety and Depression Scale; CAGE, problem drinking questionnaire; GHQ, General Health Questionnaire

^a^Van Santvoort et al. (2014)

In total, 173 COPMI families and 190 non-COPMI families completed the questionnaires at baseline. After excluding families that did not meet our criteria based on the completed questionnaires, 139 families with a parent with a mental illness and 127 families without a parent with a mental illness were included at time 1. Two years later, at time 2, 125 of the COPMI completed the questionnaires again. In some families, the parent with mental illness was not able or willing to complete the questionnaire, in which case the other parent did. These families (n = 12) were excluded from the current analyses. Of the non-COPMI families, 123 completed the questionnaires at both measurement points. In each group, one more family had to be excluded as the adolescents did not complete the questionnaires about internalizing and externalizing problems themselves, leaving 112 families with parental mental illness and 122 families without parental mental illness in the present study.

### Measures

#### Adolescent Coping Strategy (T1)

To assess coping, the Dutch Utrecht Coping List for Adolescents (UCL-A; Bijstra et al. 1994) was used. Items were measured on a 4-point scale ranging from (1) rarely or never to (4) very often. In the present study, the subscales ‘confrontation’ and ‘seeking social support’ were selected to represent active coping, and the subscales ‘depressive reactions’ and ‘avoidance’ were selected to represent passive coping (Meijer et al. 2002). The confrontation subscale consisted of 7 items (e.g., “When I have a problem, I think of different ways to solve the problem”, α = 0.77), the seeking social support subscale consisted of 6 items (e.g., “When I have a problem, I ask someone for help”, α = 0.86), the depressive reactions subscale consisted of 7 items (e.g., “When I have a problem, it feels like I cannot do anything about it”, α = 0.63), and the avoidance subscale consisted of 8 items (e.g., “When I have a problem, I wait to see what happens first”, α = 0.66). A sum score of each coping strategy was calculated, with higher scores indicating more use of that particular coping strategy.

#### Adolescent Self-Esteem (T1)

Global self-esteem was measured using the Rosenberg Self-Esteem Scale (RSES; Rosenberg 1965). The RSES comprises 10 items rated by adolescents on a 4-point scale ranging from (1) does not apply to me at all to (4) applies to me very well (e.g., “I feel that I have a number of good qualities”, α = 0.89). A higher score indicates higher self-esteem.

#### Adolescent Self-Disclosure (T1)

Adolescents’ disclosure towards parents was measured with an adapted version of the Self-Disclosure Index (SDI; Miller et al. 1983). The adapted version (Finkenauer et al. 2002) consists of 10 items measured on a 5-point scale ranging from (1) not at all to (5) extremely (e.g., “I share my deepest feelings with my parents”, α = 0.92). A sum score was used in the analyses, with a higher score indicating greater disclosure.

#### Perceived Family Support (T1)

Adolescents’ perceived social support from their family was measured with family subscale of the Multiple Scale of Perceived Social Support (MSPSS; Zimet et al. 1988). The family subscale consisted of 4 items rated by adolescents on a 7-point scale, ranging from (1) strongly disagree (7) to strongly agree (e.g., “My family really tries to help me”, α = 0.81). A sum score was calculated with a higher score indicating more perceived social support from the family.

#### Parental Mental Illness (T1)

Based on the criteria described in the procedure section, families were categorized into two types, those with parents without a mental illness (coded 0) or those with a parent with a mental illness (coded 1).

#### Current Parental Mental Health (T1)

Current mental health of parents was measured using a short 12-item version of the General Health Questionnaire (GHQ-12, Goldberg 1972; Goldberg and Williams 1988), which focuses on the inability to function normally as well as the appearance of new and distressing experiences. Parents rated the items of the GHQ-12 on a 4-point scale, with response choices (0) less than usual to (3) much more than usual (e.g., “Have you recently felt you couldn’t overcome your difficulties?” α = 0.94). The GHQ-12 can be recoded on a Likert type scale (0–1–2–3), with a possible range of 0–36, and a binary GHQ-scale (0–0–1–1), with a possible range of 0–12. As the GHQ manual favours the GHQ recoding, we selected this binary method for the current study. Higher scores indicate greater levels of general psychiatric distress.

#### Parental Monitoring (T1)

Parental monitoring was assessed using nine items completed by parents (Kerr and Stattin 2000). These items were measured on a 5-point Likert scale ranging from (1) “never” to (5) “often” (e.g., “Do you know who your child has as friends during his or her free time?” α = 0.82). A sum score was used in the analyses, with a high score indicating a high amount of parental monitoring.

#### Parental Support (T1)

Parental support was assessed using the Relationship Support Inventory (RSI; Scholte et al. 2001). The RSI comprises 12 items with response choices (1) absolutely untrue to (5) absolutely true. (e.g., “I show my child that I admire him/her”, α = 0.82). The sum score was used in the analyses, with a higher score indicating high parental support.

#### Family Environment (Cohesion, Expressiveness, Conflict) (T1)

The quality of the interpersonal relationship among family members was assessed using the ‘cohesion’, ‘expressiveness’, and ‘conflict’ subscales of the Dutch translation of the Family Environment Scale (FES; Moos and Moos 1986; GKS-II; Jansma and de Coole 1996) completed by parents. All three subscales consisted of 11 items requiring yes/no answers. The cohesion subscale measured the amount of support and commitment among the family members (e.g., ‘‘There is plenty of time and attention for everyone in our family’’, α = 0.64). The expressiveness subscale assessed the opportunity to express emotions and opinions openly and directly within the family (e.g., “There are many spontaneous discussions in our family”, α = 0.67). The conflict subscale assessed the expression of anger, aggression, and conflictive interactions amongst the family members (e.g., ‘‘Family members often criticize each other’’, α = 0.70). Sum scores of each subscale were calculated with higher scores indicating higher family cohesion, expressiveness, and conflict.

#### Adolescent Problem Behavior (T1 and T2)

Adolescent internalizing and externalizing problems were assessed at T1 and T2 with the Dutch version of the Youth Self Report (YSR, Achenbach 1991; Verhulst et al. 1996). The YSR measured the problems adolescents experienced in the previous 6 months. The items were rated by the adolescents on a 3-point scale ranging from (0) does not apply to me at all to (2) often applies to me. Adolescent internalizing problems were assessed with the anxious/depressed subscale, the withdrawn/depressed subscale, and the somatic complaints subscale. The anxious/depressed subscale consisted of 13 items (e.g., “I feel that no one loves me”), the withdrawn/depressed subscale had 8 items (e.g., “I am secretive or keep things to myself”), and the somatic complaints subscale 10 (e.g., “I have nightmares”). A sum score was calculated with higher scores indicating more internalizing problems (T1: α = 0.85, T2: α = 0.89). Adolescent externalizing problems were assessed with the aggressive and rule-breaking behavior subscales. The aggressive behavior subscale consisted of 17 items (e.g., “I destroy things belonging to others”), and the rule-breaking behavior subscale had 15 items (e.g., “I set fires”). A sum score was calculated, with higher scores indicating more externalizing problems (T1: α = 0.81, T2: α = 0.84).

### Statistical Analyses

The data were analyzed using the Statistical Package for the Social Sciences (SPSS) for Windows, version 20.0. Differences in demographic characteristics were examined using *t* tests and χ^2^ test. Means and standard deviations of the main variables were calculated. Changes in internalizing and externalizing behavior from T1 to T2 were tested with paired samples *t* tests using the continuous scores. Pearson correlation coefficients were calculated between the main variables. Separate regression analyses were conducted for each possible protective factor to investigate the relationship between the individual, dyadic (parent–child), and family factors (measured at T1) and internalizing and externalizing behavior (measured at T1 and T2), both cross-sectionally and longitudinally (i.e., predicting adolescent’ internalizing and externalizing behavior at T2 while controlling for the levels of internalizing and externalizing behavior of T1). In the regression analyses, we controlled for adolescent age and gender, recruitment strategy, and current parental mental health (based on the GHQ). Moreover, we examined differences in the relationships between the individual and (dyadic) family factors and adolescent problem behavior between COPMI and non-COPMI, testing moderation effects using interaction terms with parental mental illness (e.g., self-esteem*parental mental illness, parental monitoring*parental mental illness, family cohesion*parental mental illness). For single regression analyses with one predictor and four control variables, a medium effect size (R^2^) of 0.15, a *p* value of 0.05, and a power of 0.80, a minimum of 92 cases are needed. Calculations were done with G*Power 3.1 (Faul et al. 2009). The present study contained 112 cases (i.e., families with parental mental illness); the sample size of the present study was therefore sufficient.

## Results

### Descriptive Statistics

Table 2 describes the characteristics of the study population for COPMI and non-COPMI separately. Interesting differences are that one in four adolescent COPMI’s with a parent with mental illness did not live with both biological parents, which is more than in the control group. In COPMI families the parents were more often unemployed than in non-COPMI families. As expected, parents with a mental illness scored significantly higher than those without a mental illness on the GHQ, the HADS, and the CAGE. Parents reported that they had the following type of mental health problems: mood problems (45.5 %), anxiety problems (25.0 %), stress-related complaints (12.5 %), personality disorder (10.7 %), developmental disorder (7.1 %), schizophrenic/psychotic disorder (3.6 %), problems with grief/unresolved past (3.6 %), alcohol addiction (1.8 %), eating disorder (0.9 %), and relationship problems (0.9 %).

**Table 2:** Characteristics of the study population divided by families with a parent with mental illness (n = 112) and families with parents without mental illness at time 1 (n = 122)

	PMI	No PMI	Test of significance
Adolescent age	13.44 (1.43)^a^	13.77 (1.44)^a^	−1.78^d^
Adolescent gender			
Female	56 (50.0)^b^	57 (46.7)^b^	0.25^c^
Male	56 (50.0)^b^	65 (53.3)^b^	
Adolescent living situation			
With both parents	84 (75.0)^b^	106 (86.9)^b^	5.40*^c^
Other (e.g., mother only)	28 (25.0)^b^	16 (13.1)^b^	
Parental age	44.92 (5.29)^a^	45.88 (4.53)^a^	−1.49^d^
Parental gender			
Female	83 (74.1)^b^	94 (77.0)^b^	0.27^c^
Male	29 (25.9)^b^	28 (23.0)^b^	
Parental employment status			
At least one parent employed	94 (83.9)^b^	119 (97.5)^b^	13.25***^c^
Both parents unemployed	18 (16.1)^b^	3 (2.5)^b^	
Parental mental health			
GHQ	5.06 (4.12)^a^	0.18 (0.39)^a^	12.50***^d^
HADS-A	8.63 (4.13)^a^	2.99 (1.84)^a^	13.30***^d^
HADS-D	6.73 (4.56)^a^	1.24 (1.62)^a^	12.08***^d^
CAGE	0.67 (1.06)^a^	0.19 (0.39)^a^	3.58**^d^

PMI, parent with mental illness; no PMI, no parents with mental illness; GHQ, General Health Questionnaire; HADS-A, subscale Anxiety of the Hospital Anxiety and Depression Scale; HADS-D, subscale Depression of the Hospital Anxiety and Depression Scale; CAGE, problem drinking questionnaire

* *p* < 0.05, ** *p* < 0.01, *** *p* < 0.001

^a^Values represent mean (SD)

^b^Values represent n (%)

^c^Values represent χ^2^ statistic

^d^Values represent *t* value statistic

Means and standard deviations of the main study variables are outlined in Table 3, for adolescent COPMI and non-COPMI separately. Adolescent COPMI were more likely to show depressive reactions as coping strategy and had lower self-esteem than adolescent non-COPMI. In addition, parents with a mental illness reported less parental monitoring and support than parents without a mental illness. Furthermore, in COPMI families, the family environment was more negative (i.e., less cohesive, less expressive, more conflicting, and less supportive) than in non-COPMI families. Adolescent COPMI reported more internalizing and externalizing problems than adolescent non-COPMI, both at time 1 and at time 2.

**Table 3:** Means and standard deviations of main variables divided by families with a parent with mental illness (n = 112) and families with parents without mental illness at time 1 (n = 122)

	PMI	No PMI	*t* Test
Individual factors (T1)			
Coping: confrontation	15.25 (3.46)	15.20 (3.50)	0.11
Coping: seeking social support	13.29 (4.07)	14.08 (3.84)	−1.54
Coping: depressive reaction pattern	11.11 (2.78)	10.35 (2.77)	2.09*
Coping: avoidance	16.59 (3.10)	15.79 (3.61)	1.80
Self-esteem	22.04 (6.07)	24.05 (5.15)	−2.71**
Dyadic factors (T1)			
Parental monitoring	41.00 (3.92)	42.51 (3.11)	−3.24**
Parental support	51.23 (5.80)	53.47 (4.43)	−3.28**
Self-disclosure	32.96 (8.21)	34.61 (8.13)	−1.53
Family factors (T1)			
Family cohesion	8.31 (1.92)	9.03 (1.50)	−3.18**
Family expressiveness	8.31 (2.28)	9.57 (1.46)	−4.95***
Family conflict	4.55 (2.29)	3.70 (2.39)	2.80**
Perceived family support	22.52 (5.26)	23.94 (4.69)	−2.16*
Adolescent outcomes (T1)			
Internalizing problems	10.70 (6.74)	8.06 (5.96)	3.18**
Externalizing problems	9.07 (5.57)	7.51 (5.18)	2.22*
Adolescent outcomes (T2)			
Internalizing problems	9.76 (7.74)	7.53 (7.30)	2.26*
Externalizing problems	7.85 (5.98)	6.14 (5.28)	2.32*

PMI, parent with mental illness; no PMI, no parents with mental illness

* *p* < 0.05, ** *p* < 0.01, *** *p* < 0.001

Examining the change in problem behavior over time, no significant differences between mean levels of internalizing problems were found between time 1 and time 2 for adolescent COPMI (t(111) = 1.53, *p* = 0.128). For externalizing problems, however, adolescent COPMI reported significantly fewer problems over time (t(111) = 2.10, *p* = 0.038).^1^ Similar results were found for adolescent non-COPMI, indicating non-significant differences between mean levels of internalizing problems over time (t(121) = 1.01, *p* = 0.313). In this group too, a significant decrease in externalizing problems was found (t(121) = 3.66, *p* < 0.001).

### Correlations Between Individual, Dyadic (Parent–Child), and Family Protective Factors and Problem Behavior

Table 4 presents Pearson correlations for all study variables. In families with parental mental illness, active coping strategies (i.e., confrontation and seeking social support) were not related to internalizing and externalizing problems at time 1. Confrontation coping was moderately and negatively related to internalizing problems at time 2. Depressive reaction pattern had a moderate to strong association with both internalizing and externalizing problems at time 1 and time 2. Avoidant coping was related only to internalizing problems at time 1. Self-esteem was negatively related to internalizing problems and externalizing problems at time 1. Parental monitoring was negatively and moderately related to externalizing problems at time 1 and to both internalizing and externalizing problems at time 2. Parental support was negatively related to externalizing problems at time 2. Self-disclosure towards parents was negatively related to internalizing problems at time 1 and time 2, and to externalizing problems, albeit only at time 2. No significant correlations were found between family environment (i.e., cohesion, expressiveness, conflict) and problem behavior. Perceived family support was negatively related to internalizing problems.

**Table 4:** Pearson correlations between protective factors and problem behavior

Measure	1	2	3	4	5	6	7	8
1. Confrontation T1	–	0.51**	−0.03	0.04	0.34**	0.09	0.18	0.38**
2. Seeking social support T1	0.31**	–	0.01	−0.10	0.32**	0.01	0.19	0.52**
3. Depressive reaction T1	−0.05	0.07	–	0.27**	−0.49**	−0.27**	0.05	−0.32**
4. Avoidance T1	0.19*	−0.01	0.47**	–	−0.16	−0.11	−0.00	−0.05
5. Self-esteem T1	0.34**	0.30**	−0.44**	−0.32**	–	0.15	0.05	0.46**
6. Parental monitoring T1	0.04	0.03	−0.04	0.05	0.04	–	0.41*	0.24*
7. Parental support T1	0.05	0.18*	−0.09	0.10	0.12	0.35**	–	0.19
8. Self-disclosure T1	0.30**	0.43**	−0.16	−0.01	0.36**	0.24**	0.41**	–
9. Family cohesion T1	−0.05	−0.12	−0.01	−0.12	−0.03	0.18*	0.23*	0.10
10. Family expressiveness T1	−0.05	0.08	−0.03	−0.09	0.01	0.18*	0.33**	0.13
11. Family conflict T1	−0.03	−0.04	−0.01	−0.07	−0.01	−0.02	−0.21*	−0.23*
12. Perceived family support T1	0.28**	0.38**	−0.16	−0.02	0.43**	0.08	0.22*	0.62**
13. Internalizing problems T1	−0.17	−0.15	0.58**	0.38**	−0.68**	−0.04	−0.06	−0.26**
14. Externalizing problems T1	−0.07	−0.05	0.32**	0.17	−0.23*	−0.26**	−0.09	−0.25**
15. Internalizing problems T2	−0.22*	−0.16	0.43**	0.19*	−0.57**	−0.01	−0.05	−0.19*
16. Externalizing problems T2	−0.07	0.05	0.20*	−0.00	−0.11	−0.18*	−0.08	−0.13

Measure	9	10	11	12	13	14	15	16
1. Confrontation T1	−0.12	0.02	0.06	0.31**	−0.11	−0.03	−0.24*	−0.16
2. Seeking social support T1	−0.16	0.10	−0.09	0.34**	−0.08	0.06	−0.11	−0.08
3. Depressive reaction T1	−0.05	0.01	0.12	−0.22*	0.64**	0.39**	0.40**	0.25**
4. Avoidance T1	−0.03	−0.12	0.03	−0.04	0.27**	0.13	0.06	−0.07
5. Self-esteem T1	0.01	0.10	−0.01	0.34**	−0.60**	−0.22*	0.14	0.02
6. Parental monitoring T1	0.25**	0.33**	−0.22*	0.17	−0.08	−0.31**	−0.25**	−0.30**
7. Parental support T1	0.34**	0.46**	−0.39**	0.16	0.09	−0.13	0.02	−0.21*
8. Self-disclosure T1	−0.05	0.11	−0.12	0.51**	−0.28**	−0.15	−0.37**	−0.24*
9. Family cohesion T1	–	0.31**	−0.29**	0.04	−0.10	−0.13	−0.03	−0.00
10. Family expressiveness T1	0.17	–	−0.18	0.14	0.01	0.05	0.02	−0.01
11. Family conflict T1	−0.25**	0.05	–	−0.04	0.17	0.07	0.14	0.02
12. Perceived family support T1	0.19*	0.01	−0.23*	–	−0.22*	−0.17	−0.25**	−0.18
13. Internalizing problems T1	−0.08	0.09	0.09	−0.33**	–	0.33**	0.61**	0.26**
14. Externalizing problems T1	−0.24**	−0.08	0.24**	−0.20*	0.44**	–	0.24*	0.43**
15. Internalizing problems T2	−0.10	0.07	0.13	−0.30**	0.65**	0.23**	–	51**
16. Externalizing problems T2	−0.28**	−0.04	0.26**	−0.26**	0.26**	0.69**	0.38**	–

Coefficients above the diagonal are for families with a parent with mental illness (n = 112); coefficients below the diagonal are for families with parents without mental illness (n = 122)

* *p* < 0.05, ** *p* < 0.01 (2-tailed)

### The Relationship Between Individual, Dyadic, and Family Factors and Problem Behavior at Baseline

Cross-sectional analyses, in which adolescent gender, adolescent age, recruitment strategy, and current parental mental health were controlled for, showed that passive coping strategy was positively related to internalizing problems, and self-esteem and self-disclosure were negatively related to internalizing problems (see Table 5). Active coping strategy, parental monitoring, parental support, and the broader family factors (i.e., family cohesion, family expressiveness, family conflict, and perceived family support) were not related to internalizing problems. Depressive reaction pattern was positively related to externalizing problems while self-esteem and parental monitoring were negatively related to externalizing problems. Active coping strategy, avoidant coping strategy, parental support, self-disclosure, and the broader family factors (i.e., family cohesion, family expressiveness, family conflict, and perceived family support) were not related to externalizing problems.

**Table 5:** Separate regression analyses (cross-sectional and longitudinal) examining one predictor of internalizing and externalizing problems separately for adolescents who have a parent with mental illness (n = 112)

Predictors T1	Internalizing problems				Externalizing problems			
	T1		T2		T1		T2	
	∆*R* ^2^	β	∆*R* ^2^	β	∆*R* ^2^	β	∆*R* ^2^	β
Step 1								
Control variables^a^	0.17**		0.39***		0.07		0.27***	
Step 2^b^								
Confrontation	0.00	−0.06	0.03*	−0.17*	0.00	0.01	0.01	−0.11
Seeking social support	0.01	−0.11	0.01	−0.08	0.00	0.05	0.02	−0.13
Depressive reaction	0.30***	0.57***	0.00	0.02	0.14***	0.39***	0.00	0.04
Avoidance	0.09***	0.31***	0.01	−0.10	0.03	0.17	0.01	−0.09
Self-esteem	0.24***	−0.51***	0.00	−0.0.04	0.04*	−0.20*	0.00	−0.03
Parental monitoring	0.00	−0.04	0.04*	−0.20*	0.06*	−0.25*	0.02	−0.16
Parental support	0.03	0.18	0.00	0.00	0.01	−0.07	0.01	−0.09
Self-disclosure	0.05*	−0.23*	0.04*	−0.20*	0.01	−0.10	0.02	−0.15
Family cohesion	0.00	−0.04	0.00	0.05	0.01	−0.10	0.01	0.11
Family expressiveness	0.00	0.07	0.00	0.04	0.02	0.14	0.00	0.01
Family conflict	0.00	0.07	0.00	0.01	0.01	0.09	0.01	−0.08
Perceived family support	0.02	−0.15	0.01	−0.10	0.02	−0.14	0.00	−0.05

* *p* < 0.05, ** *p* < 0.01, *** *p* < 0.001

^a^Control variables included age, gender, recruitment strategy, and severity of parental mental illness in the cross-sectional analyses. Additional control variables included internalizing and externalizing problems at time 1 in the longitudinal analyses

^b^Beta and R^2^ were calculated in separate regression analyses for each predictor

### The Protective Effect of Individual, Dyadic (Parent–Child), and Family Factors on the Development of Problem Behavior

Longitudinal analyses showed that, when controlling for adolescent gender, adolescent age, recruitment strategy, current parental mental health, and problem behavior at time 1, a more confronting coping strategy, high parental monitoring, and greater child disclosure predicted fewer internalizing problems 2 years later (see Table 5). Seeking social support as coping strategy, a passive coping strategy, self-esteem, parental support, and the broader family factors (i.e., family cohesion, family expressiveness, family conflict, and perceived family support) did not protect adolescents against developing internalizing problems over time. No protective factors were found for externalizing problems.

### Differences in Protective Factors Between Adolescent COPMI and Non-COPMI

No significant interaction effects were found for internalizing and externalizing problems in the cross-sectional analyses, indicating that parental mental illness did not moderate the relationship of the possible individual and (dyadic) family factors with adolescent internalizing and externalizing problems. The longitudinal analyses revealed only one significant finding, namely that the interaction between family cohesion and parental mental illness predicted externalizing problems at time 2 (β = 0.13, *p* = 0.017), indicating a significant moderation effect of family cohesion. Separate regression analyses for families with and without parental mental illness showed that family cohesion predicted externalizing problems 2 years later only for families without a parent with mental illness (β = −0.26, *p* = 0.003) and not for those with a parent with mental illness (β = −0.10, *p* = 0.296).

## Discussion

The main aim of the present study was to test the factors that could protect adolescents who have a parent with mental illness against developing internalizing and externalizing problems. Our analyses revealed that the less adolescents used passive coping strategies, the higher their self-esteem, and the more they disclosed information to their parents, the fewer internalizing problems they reported at baseline. Greater self-disclosure also predicted fewer internalizing problems over 2 years, as did more use of active coping strategies and higher parental monitoring. For externalizing problems, a more passive reaction pattern, higher self-esteem, and more parental monitoring were related to fewer problems. None of these factors however predicted changes in externalizing problems over time. No differences were found between families with and without parental mental illness in the relationships between protective factors and problem behavior.

As expected and consistent with previous research (e.g., Beardslee et al. 2011), adolescent COPMI reported more internalizing and externalizing problems than adolescent non-COPMI, both at baseline and at follow-up. Neither groups of adolescents showed a significant change in internalizing problems over time. A possible explanation could be that we included both boys and girls in the analyses, where others found a slight increase in problem behavior for girls and a slight decrease for boys (Bongers et al. 2004). All adolescents (regardless of parental mental illness) reported significantly fewer externalizing problems 2 years later. This is in line with previous research, which showed that aggressive behavior declined over the course of adolescence, that rule-breaking behavior increased slightly over time, and externalizing problems in general decreased over time (Bongers et al. 2004; Stanger et al. 1997).

### Possible Protective Factors and Internalizing Problems

Of the individual factors, coping and self-esteem seemed to play a role in internalizing problems. In general adolescent populations, it has been documented that high self-esteem is related to less problem behavior (Donnellan et al. 2005; Dumont and Provost 1999). The present study examined this in adolescent COPMI and found a similar relationship. However, self-esteem did not seem to predict changes in internalizing problems over time. A possible explanation is that the causal relationship could be the other way around. That is, having more internalizing problems might lower self-esteem over time rather than the reverse (e.g., Rosenberg et al. 1989). At baseline, using less passive coping strategies seemed to be related to positive outcomes (i.e., fewer internalizing problems) for adolescent COPMI, which is in line with previous studies conducted with the general adolescent population (Dumont and Provost 1999). Interestingly, using an active rather than a passive coping strategy to deal with problems (e.g., trying to find a solution) seemed to protect adolescent COPMI against developing feelings of anxiety or depression and somatic complaints. This effect has not previously been examined in adolescents who have a parent with mental illness. This finding suggests that focusing on enhancing active coping strategies could be relevant for preventive interventions.

Looking at dyadic (parent–child) factors in internalizing problems, both adolescent’s self-disclosure to parents and parental monitoring seem to be important. The more adolescents disclosed information about themselves to their parents reported, the fewer internalizing problems. Self-disclosure also seemed to protect adolescents against developing these problems over 2 years. Previous research in a general adolescent sample did not find a significant relationship between self-disclosure and depressive mood, but it did find a relationship between self-disclosure and physical complaints (Finkenauer et al. 2002), which were also included in our internalizing problems scale. Also, parents’ knowledge of their child’s whereabouts and who they hang out with (monitoring) also seemed to protect the at-risk youth against developing internalizing problems. Ours is the first study to reveal this relationship: more parental monitoring predicts fewer internalizing problems of adolescent COPMI. This is in line with, and adds to, the statement by Dishion and McMahon (1998) that parental monitoring could serve as a protective factor for high-risk children; they proposed that adequate parental monitoring is central to healthy parenting. Parents need to know that they should be involved in the lives of their teenage children to improve their well-being. In sum, parent–child communication seems to play an important role in preventing internalizing problems, and it would seem sensible to pay attention to it in preventive interventions for adolescent COPMI.

The broader family factors that were measured (i.e., cohesion, expressiveness, conflict, and perceived support) do not seem to play a role in preventing internalizing problems. These factors had not been studied before in families with a broader range of parental mental illnesses (only in families with a substance-using parent; Farrell et al. 1995 or a depressed parent; Fendrich et al. 1990). In contrast to cross-sectional research with general adolescent samples (e.g., Cole and McPherson 1993), we found no (prospective) relationships between these family factors and internalizing problems. So, although factors on the dyadic parent–child level did play a role, factors on a broader family level did not relate to problem behavior.

Based on our study, the implications seem to be that focusing on active coping styles and parent–child interaction seems useful in interventions aimed at preventing internalizing problems. However, more studies are needed to replicate and extend the present results before strong conclusions can be drawn.

### Possible Protective Factors and Externalizing Problems

Regarding the relationship of individual factors with externalizing problems of adolescent COPMI, we found that the same factors as for internalizing problems played a role: less use of ‘depressive reaction pattern’ as a passive coping strategy and higher self-esteem. The finding that a passive coping strategy is related to negative outcomes is consistent with previous research (Meijer et al. 2002). The relationship we found between higher self-esteem and fewer externalizing problems was also found in previous research conducted with general adolescent samples (e.g., Donnellan et al. 2005). No individual factors seemed to predict a decrease in externalizing problems over the 2-year period from baseline to follow-up. It is possible that these causal relationships are in the opposite direction, with more externalizing problems predicting lower self-esteem and more frequent use of a passive coping strategy. This could be addressed in future research.

The results at the dyadic level revealed that only more parental monitoring was related to fewer externalizing problems of adolescent COPMI, which is in line with previous research with general adolescent samples (Jacobson and Crockett 2000; Patterson 1993). Unlike with internalizing problems, no (prospective) relationship was found between self-disclosure and externalizing problems. Just like with the individual factors, also none of the dyadic factors seemed to protect adolescent COPMI against developing aggressive and rule-breaking behavior over time.

No significant (prospective) relationships were found between the broader family factors that were measured (i.e., cohesion, expressiveness, conflict, and perceived support) and externalizing problems of adolescent COPMI. Previous cross-sectional research with adolescent non-COPMI had found significant relationships between family factors and externalizing problems (e.g., Licitra-Kleckler and Waas 1993). Perhaps these family factors play a different role in the lives of adolescents without a parent with mental illness than of those with a parent with mental illness. This explanation is supported by our finding that family cohesion was only related to externalizing problems over time for adolescent non-COPMI and not adolescent COPMI.

### Differences in Protective Factors Between Adolescents With and Without a Parent with Mental Illness

Differences were found in mean levels of the individual (i.e., more depressive reaction pattern, less self-esteem), dyadic (i.e., less parental monitoring and support), and broader family (i.e., more negative family environment, less perceived family support) factors between families with a parent with mental illness and families without a parent with mental illness. Adolescent COPMI also reported more problems than adolescent non-COPMI, both at baseline and at follow-up. However, despite these differences, the relationships between the protective factors and problem behavior were similar for the two groups. The only significant difference was found for the relationship between family cohesion and externalizing problems at follow-up. We should be aware that, since we tested a large number of interactions, this finding could be based on chance. The fact that we found virtually no moderation effects when we compared the relationships of possible protective factors and problem behavior for COPMI and non-COPMI seem to indicate that the interventions that are used in the general adolescent population to help them with problem behavior could also be used for adolescents with a parent with mental illness. However, as adolescent COPMI do show more problem behavior, they seem to be more in need of these interventions.

## Limitations, Strengths, and Future Research

This study has several limitations. First, it used only self-report measures. Future studies should use multi-method designs that would include observations to examine the factors at the dyadic (parent–child) and family level (Holmbeck et al. 2002). Next, some of the variables in the tested relationships were completed by the adolescents only (i.e., the relationships between individual factors, self-disclosure, perceived family support, and the outcome measures). Thus, by using a single informant, the cognitive characteristics and personality of adolescents in our study might have accounted for significant relationships between variables rather than between true score variance (e.g., Youngstrom et al. 1999). Future research could control for this shared rater bias. On the other hand, we did use multiple informants for several other relationships examined in the present study (i.e., the relationships between parental support, parental monitoring, family environment and the outcome measures). Another limitation is that we performed a large number of separate regression analyses. When performing multiple analyses, results should be interpreted cautiously as these results could have been due to chance. Furthermore, other variables could function as protective factors against developing problem behavior of these adolescents, such as knowledge about parental mental illness. However, this study did capture a broad range of potential predictors at an individual, a dyadic, and a broader family level that had been found to be related to and/or predict problem behavior in the general adolescent population. Other strengths are the focus on an understudied sample, that is: adolescents who have a parent with mental illness and the inclusion of a control group to explore whether the relationships we found would be different between these adolescents and adolescents in general, regardless of parental psychological problems.

## Practical Implications

Our results indicate that it seems important to include training active coping strategies in preventive interventions. Fortunately, enhancing coping skills is already included in some preventive interventions, such as in the Adolescent Coping with Stress Course, which is a group cognitive intervention for preventing depression in adolescents with a depressed parent (Clarke et al. 2001; Garber et al. 2009). Another practical implication of our findings is that preventive interventions for adolescent COPMI might benefit from focusing on parent–child communication. The existing interventions for families with a parent with mental illness already include this to some extent, but the focus there is on communicating with parents about their mental illness (e.g., Family Talk Intervention, Beardslee et al. 1996; Let’s Talk about Children Intervention; Solantaus and Toikka 2006). The results of the current study imply that it is not only important to teach parents how to talk to children about the mental illness, but also how to talk with their children about general topics. For parents, it is important to know where their child is and who they hang out with, for instance, and at the same time, it is important for adolescents to tell their parents what is on their mind. To our knowledge, no particular attention has yet been paid to parental monitoring and adolescent self-disclosure. Therefore, intervention developers could include parts of existing general parenting programs when considering this specific at-risk group. For example, Parent Management Training (PMT; e.g., Kazdin 1997) and the Incredible Years (Webster-Stratton and Reid 2003) already address parental monitoring to prevent child problems. Overall, the results of the present study imply that it would be useful to combine teaching youth active coping strategies with parent–child communication about everyday life of both parents and adolescents (i.e., parental monitoring and adolescent self-disclosure in specific) in a family-based intervention for families in which a parent has a mental illness in order to prevent internalizing problems.

## Footnotes

1 Adolescents who were recruited via the previous study (n = 16; Van Santvoort et al. 2014), and who had therefore already received a preventive intervention, did not differ from those recruited via other recruitment strategies (n = 96) on Internalizing and Externalizing problems at Time 1 and Time 2, nor on changes in problem behavior over time (*p* > 0.29).
